# The Effect of Hamatum Curvature Angle on Carpal Tunnel Volumetry: A Mathematical Simulation Model

**DOI:** 10.1155/2020/7582181

**Published:** 2020-03-10

**Authors:** Nazmi Bülent Alp, Tufan Kaleli, Onur Can Kalay, Fatih Karpat, Gokhan Akdag, Aslı Ceren Macunluoglu, Gamze Saygı Oral

**Affiliations:** ^1^Department of Orthopedics and Traumatology, Bursa Uludag University School of Medicine, Bursa, Turkey; ^2^Department of Mechanical Engineering, Bursa Uludag University, Bursa, Turkey; ^3^Istanbul Beylikdüzü State Hospital, Orthopedics and Traumatology Department, Istanbul, Turkey; ^4^Department of Biostatistics, Uludağ University, Institute of Health Sciences, Bursa, Turkey; ^5^Independent Researcher, Istanbul, Turkey

## Abstract

In carpal tunnel volume measurements, the angle of the hamatum curvature is not considered a variable, and its effect on carpal tunnel volume has not been investigated. We hypothesize that a change in the anatomical angle of the hamatum curvature changes the carpal tunnel volume. To prove our hypothesis, we used a mathematical simulation model considering the carpal tunnel as a truncated cone. We reviewed the wrist CT scans of 91 adults (>18 years of age), including 51 men and 40 women. We measured the angle of the hamatum curvature in the CT scans. We measured cross-sectional areas at the outlet of the carpal tunnel at the level of the trapezium and hook of hamate (*r*1) and at the inlet at the level of the scaphoid and pisiform (*r*2) and the length (*h*) of the carpal tunnel. We attempted to calculate the effect of 2 degree by 2-degree changes in the angle of the hamatum curvature between the angles of 98° and 140° on the carpal tunnel volume. The mean angle of the hook of hamatum of the subjects was 122.55° ± 8.20° (range, 97.20° − 139.31°). No suitable cutoff point was found for the angle values. There was no difference between the gender groups according to the angle value. The data clearly show that there is a high correlation between carpal tunnel volume and the angle of hamatum curvature. The results of our study emphasize the importance of taking into account the anatomical features of the hamatum bone, especially the angle of curvature, which may play a predisposing role in idiopathic carpal tunnel syndrome.

## 1. Introduction

Carpal tunnel syndrome (CTS) is the most common peripheral compression neuropathy syndrome in the USA [1]. A carpal tunnel is a fibro-osseous tunnel where its inner boundaries are made up of soft tissues and carpal bones. The transverse carpal ligament (TCL) forms the volar margin of the tunnel. Proximally, the TCL attaches to the scaphoid tuberosity and pisiform; distally, it attaches to the hook of hamate and the ridge of the trapezium. The hook of hamate is a reliable landmark [2, 3] in hand surgery, especially in carpal tunnel release [4, 5, 6]. Some studies have reported a small number of bipartite, hypoplastic, and aplastic hooks of the hamate [2–12]. In another study, Huang et al. measured 2000 hamates, and they classified 3.1% (62/2000) as hypoplastic and aplastic. The volume of the carpal tunnel is only slightly less than the volume of the soft tissue content [13]; therefore, any process that reduces the volume of the carpal tunnel may cause compression of the median nerve. We believe that anatomical changes in the hamate bone may alter the volume of the carpal tunnel due to a change in the ulnar border. Various authors investigated the volume and cross-sectional areas of the carpal tunnel by computed tomography, magnetic resonance imaging, silicon mold casting, and ultrasound [14–16]. To the best of our knowledge, the relationship between the shape of the hamatum curvature or, in other words, the angle of the curvature and its effects on carpal tunnel volume has never been studied in the literature.

The aim of this study was two-fold: (a) to study the angle of the hamatum curvature and whether there is any gender difference and (b) to evaluate the relationship between the angle of the hamatum curvature and carpal tunnel volume using a mathematical simulation model.

## 2. Materials and Methods

**Figure 1:**
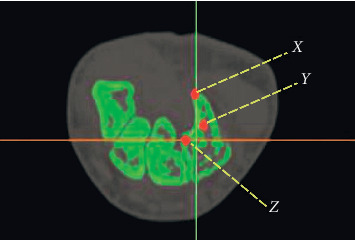
Three points were identified locally to measure the angle of hamatum curvature. *X*, tip of hamate; *Y*, hamate curvature; *Z*, base of hamate.

**Figure 2:**
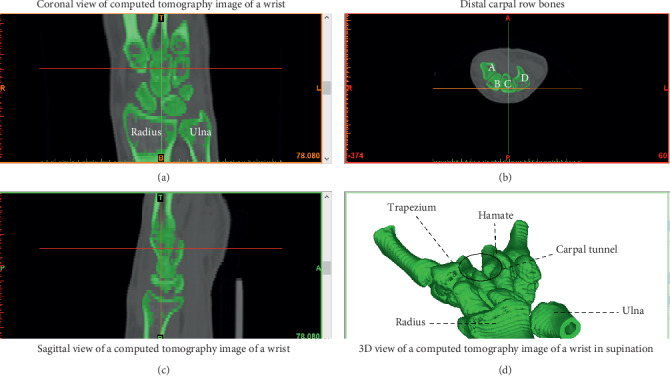
Measurements were conducted with MIMICS 20.0 toolboxes. (a) Coranal view of computed tomography image of a wrist. (b) Distal carpal row bones. (c) Sagittal view of a computed tomography image of a wrist. (d) 3D view of a computed tomography image of a wrist in supination.

**Figure 3:**
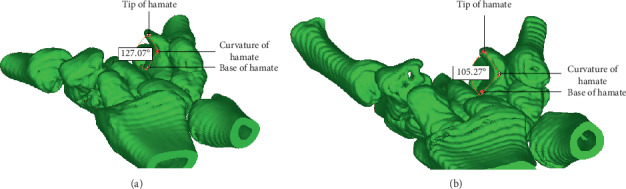
Measurement of angle of hamatum curvature.

The current study was approved by the Hospital's Ethics Committee. We reviewed the wrist CT scans of 91 adults (>18 years of age), including 51 men and 40 women. Exclusion criteria were scans of inadequate quality to make reliable measurements, carpal abnormalities, and evidence of previous hamatum fracture, malunion, or pseudoarthrosis. For the first part of the present study, three points were identified locally to measure the angle of the hamate hook from CT scan data (Figure 1). Measurements were conducted with MIMICS 20.0 toolboxes (Figures 2 and 3(a), 3(b)).

**Figure 4:**
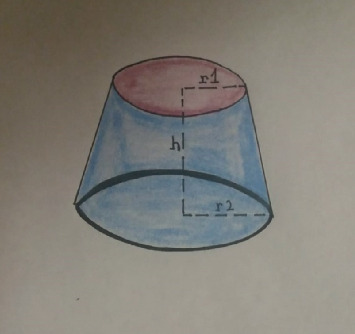
Schematic representation of a truncated cone.

For the second part of the study, we hypothesized that changing the angle of the hamatum curvature changes the volume of the carpal tunnel. To prove our hypothesis, we used a mathematical simulation model considering the carpal tunnel as a truncated cone (Figure 4).

**Figure 5:**
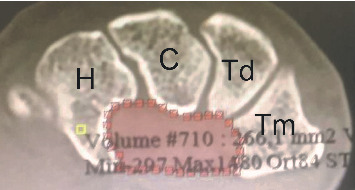
Measurement of outlet cross-sectional area.

**Figure 6:**
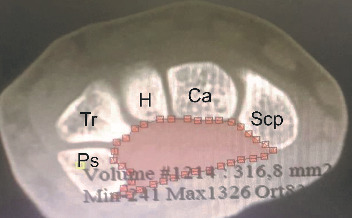
Measurement of inlet cross-sectional area.

**Figure 7:**
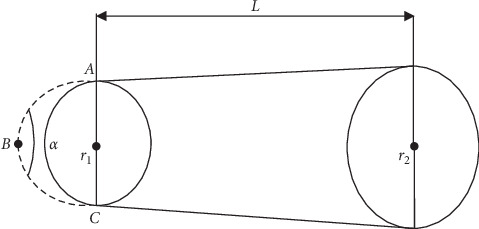
Schematic representation of a truncated cone resembling a carpal tunnel configuration with *α* representing the angle of hamatum curvature.

This model was based on a previous study by Jebson and Agee [11]. The carpal tunnel was found to have a conical shape, and the inlet was continually larger than the outlet. To the best of our knowledge, this mathematical model was used twice in the literature by Pierre-Jerome et al. and Pavlidis et al. [17, 18], and it may be considered a reliable solution for the experimental measurement of carpal tunnel volume. Only 3 values (*h*, *r*1, and *r*2) are necessary to be measured and considered a reliable method of how the changes in the angles of curvature of the hamatum are reflected in the total carpal tunnel volume. We measured cross-sectional areas at the outlet of the carpal tunnel at the level of trapezium and hook of the hamate (*r*1) (Figure 5) and the inlet at the level of the scaphoid and pisiform (*r*2) (Figure 6) and the length (*h*) of the carpal tunnel. The length of the carpal tunnel (*L*) accepted as the height of the truncated cone corresponds to the distance between the inlet and outlet of the carpal tunnel areas (Figure 7).

To estimate the carpal tunnel volume, the angular difference that occurs in the curvature of the hamatum is reduced if it changes the volume of the truncated cone . Mathematically, a change in the truncated cone volume means that the volume of the carpal tunnel is also altered. We tried to calculate how the 2 degree by 2 degree changes in the angle of hamatum curvature did reflect in the carpal tunnel volume between the lowest and the highest measured angles.

The truncated cone volume (carpal tunnel volume) formula is

(1) $$\begin{array}{c} V=\frac{1}{3}\;\pi L\left(r_{1}^{2}+r_{1}r_{2}+r_{2}^{2}\right). \end{array}$$

**Figure 8:**
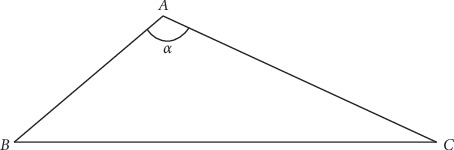
Carnot theorem (cosine theorem).

With the help of our measurements (*r*1, *r*2, and *h*), we attempt to determine how a change in the angle of the hamatum curvature affects the carpal tunnel volume. A calculation of the carpal tunnel volume is made according to the formula given above, where *V* is the carpal tunnel volume, *π* = 3.14, *h* is the height of the truncated cone, which represents the distance between the inlet and outlet of the carpal tunnel, and *r*1 and *r*2 are the outlet and inlet radii, respectively. The hook of hamate is included in the measurement of the outlet cross-sectional area, and changing the angle of the hamatum curvature will change the cross-sectional area, which will be reflected in the volume of the carpal tunnel. For a better understanding, we consider the ABC triangle below and perform calculations as follows (Figure 8).

(2) $$\begin{array}{c} c^{2}=a^{2}+b^{2}-2ab\;\cos\;\alpha . \end{array}$$

**Figure 9:**
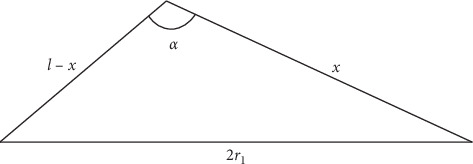
Model 1.

If we use the Carnot theorem in our model 1 (Figure 9),

(3) $$\begin{array}{c} x^{2}+{\left(l-x\right)}^{2}-2x\left(l-x\right)\cos\;\alpha ={\left(2r_{1}\right)}^{2}\\ =x^{2}+l^{2}-2lx+x^{2}-2lx\cos\;\alpha +2x^{2}\cos\;\alpha \\ =2x^{2}+2x^{2}\cos\;\alpha -2lx-2lx\cos\;\alpha +\;l^{2}\\ =2x^{2}\left(1+\cos\;\alpha \right)-2lx\left(1+\cos\;\alpha \right)+\;l^{2}\\ =\left(1+\cos\;\alpha \right)\left[2x^{2}-2lx\right]+l^{2}\\ =\left(1+\cos\;\alpha \right)2x\left[x-l\right]+l^{2}={\left(2r_{1}\right)}^{2}. \end{array}$$

We accept that there is no change in bone length and that only the *a* value is changed; therefore, *r*1 is changed. In this case, in the equation that we present above, we think that *l* is approximately 2*x*; hence, the above equation is rewritten as

(4) $$\begin{array}{c} =\left(1+\cos\;\alpha \right)2x\left(x-2x\right)+4x^{2}\\ =\left(1+\cos\;\alpha \right)\left(-2x^{2}\right)+4x^{2}\\ =2x^{2}\left[-1-\cos\;\alpha +2\right]\\ =2x^{2}\left[1-\cos\;\alpha \right]. \end{array}$$

With the help of the measured value *α*=98°, we will calculate this part and use this value for the other α values. In this case, the constant *B* can be used for the 2*x*^2^ value.

(5) $$\begin{array}{c} B=2x^{2},\\ {\left(2r_{1}\right)}^{2}=\left(1-\cos\;\alpha \right)B,\\ r_{1}=\frac{1}{2}\sqrt{B\left(1-\cos\;\alpha \right)}, \end{array}$$

For *α*=98°, *A*_1_=216, *L*=19.4, and *A*_2_=304,

(6) $$\begin{array}{c} A_{1}=\pi r_{1}^{2}⟶r_{1}=\sqrt{\frac{A1}{\pi }}=8.29\text{ changes depending on }\alpha ,\\ A_{2}=\pi r_{2}^{2}⟶r_{2}=\sqrt{\frac{A2}{\pi }}=9.83\text{ remains constant.} \end{array}$$

We then find the value of the constant *B* for = 98°,

(7) $$\begin{array}{c} {\left(2r_{1}\right)}^{2}=\left(1-\cos\;\alpha \right)B,\\ r_{1}=8.29,\\ \alpha =98^{{}^{\circ}}=1.71\text{ radian,}\\ \cos\;\left(\alpha \right)\left(1.71\text{ radian,}\right)=-0,139,\\ \;{\left(2r_{1}\right)}^{2}\;=\left(1-\left(-0.139\right)\right)B,\\ {\left(2\times 8.29\right)}^{2}\;=1.139B,\\ B=241.34. \end{array}$$

Elements of the table are as follows:

(8) $$\begin{array}{c} \alpha =98^{{}^{\circ}}-\;140^{{}^{\circ}}\;2^{{}^{\circ}\prime }\text{ angle values,}\\ X_{\text{rad}}=\text{trigonometric values converted into radian}\\ \text{ values regarding excel equations,}\\ \text{Cos }\left(\text{Alfa}\right)=\text{ cosine of the angle,}\\ r1=\text{truncated cone outlet radius,}\\ r2=\text{truncated cone inlet radius,}\\ L=\text{height of truncated cone, }\\ \text{volume}=\text{truncated cone volume.} \end{array}$$

### 2.1. Statistical Analysis

The Shapiro–Wilk test was used to determine the normal distributions of the variables. Continuous variables were expressed as median (minimum: maximum) or mean ± standard deviation (minimum: maximum), and categorical variables were expressed as *n* (%). The Mann–Whitney *U* test was used for comparisons between the groups according to the normality test results. Two paired *t*-tests were used for statistical comparison. For statistical analysis, SPSS (IBM Corp. Released 2012. IBM SPSS Statistics for Windows, Version 21.0. Armonk, NY: IBM Corp.) was used, and *p* < 0.05 was considered statistically significant.

## 3. Results

The median age of the patients who participated in the study was 34 years (10 : 88); 40 (43.96%) of the patients were female, and 51 (56.04%) were male. The angle between the identified points was measured via program toolboxes. All measurements were repeated 3 times, and the arithmetic mean was taken as the final value. The minimum and maximum angular values measured ranged from 98° to 139°.

The mean hook of the hamatum angle of the subjects was 122.55° ± 8.20° (97.20°: 139.31°). In this study, no suitable cutoff point was found for the angle values. There was a difference between gender groups according to the age distribution. The median age of women was higher than that of men (*p*=0.012).

There was no difference between the gender groups according to the angle value (*p*=0.876). For an increase in the angle of the hook of hamate 2 degrees by 2 degrees from 98 degrees to 140 degrees, a significant difference was found between the change in the angle of the hook of hamate and total carpal tunnel volume according to correlation analysis (*p* < 0.01) (Table 1).

## 4. Discussion

Some studies showing the relationship between carpal tunnel syndrome and the hamatum bone are found in the literature. Chow et al. believed that the variations in the hook of hamate (bipartite, hypoplastic, aplastic) may decrease the volume available in the carpal tunnel because of the loss of a portion of the ulnar border. [19] Moreover, they concluded that there was a significant increase in the incidence of variation in the hook of hamate in a group with CTS. A study published in 1997 by Richards and Bennet [2] evaluated the abnormalities of the hook of hamate in 131 hands. They identified 3 aplastic, 1 bipartite, and 1 nonunion of a fracture of the hook of the hamate. Their study found a significantly higher incidence (2.3%) of variations in the hook of hamate in patients presenting with CTS. However, to the best of our knowledge, the relationship between the angle of the hamatum curvature and the volume of the carpal tunnel has not been investigated in the literature to date.

The shape and dimensions of the wrist have been implicated as predisposing factors producing a narrower carpal tunnel that is more prone to increased pressure [20, 21].

Anthropometric comparison of patient and control groups indicates that wrist and hand dimensions may contribute to idiopathic carpal tunnel syndrome [22]. Various volumetric methods were used to calculate the volume of the carpal tunnel. Thin-section MRI or CT scans (4–5 mm) were used to create a 3-dimensional reconstruction image of the carpal tunnel [17, 23]. Richman et al. [23] determined the carpal tunnel volume and carpal arch within 10 cadaveric specimens using 3-dimensional computer reconstruction of magnetic resonance images. MRI-acquired data were compared with direct measurements of cadaveric carpal tunnels using the silicone injection technique. In carpal tunnel volume measurements, the angle of hamatum curvature was not considered a variable, and its effect on carpal tunnel volume was not investigated. We hypothesize that an anatomical angle change in the hamatum curvature changes the carpal tunnel volume. We tested the hypothesis of the hamatum curvature with the mathematical model. The data clearly show that there is a high correlation between the carpal tunnel volume and angle of hamatum curvature. This correlation may be significant in the development of CTS. This has, however, not been studied, as none of the subjects in this study had clinical evidence of CTS.

There are weaknesses in this study. Our measurements were performed only in healthy subjects. Because we did not know whether the hamatum curvature angle influenced the volume of the carpal tunnel before the study, we did not have a computed tomography scan in patients with carpal tunnels. We know that computed tomography will create an unnecessary radiation burden on patients. In our future studies, we aim to compare the values of the hamatum curvature angle of the healthy population with those of patients with a carpal tunnel diagnosis.

Additionally, it is evident that this mathematical model cannot simulate every wrist at 100%. In real-life cases, inlet and outlet areas are not perfect cones or circles; however, this model still provides valuable data for further clinical studies.

The results of our study will emphasize the importance of taking into account the anatomical features of the hamatum bone, especially the angle of curvature, which may play a predisposing role in idiopathic carpal tunnel syndrome. Investigation of the correlation between the angle of hamatum curvature and carpal tunnel volume requires further clinical study.

## Figures and Tables

**Table 1:** Analyses of carpal tunnel volume changes with angle differences.

Alfa	Alfa radyan	Cos (alfa)	*r*1	*r*2	*L*	Volume
98	1.710422667	−0.139173101	8,29047701	9.8369	19.4	5016.416
100	1.745329252	−0.173648178	8.41499036	9.8369	19.4	5083.523
102	1.780235837	−0.207911691	8.53694043	9.8369	19.4	5149.859
104	1.815142422	−0.241921896	8.65629006	9.8369	19.4	5215.364
106	1.850049007	−0.275637356	8.7730029	9.8369	19.4	5279.982
108	1.884955592	−0.309016994	8.8870434	9.8369	19.4	5343.655
110	1.919862177	−0.342020143	8.99837682	9.8369	19.4	5406.326
112	1.954768762	−0.374606593	9.10696924	9.8369	19.4	5467.939
114	1.989675347	−0.406736643	9.2127876	9.8369	19.4	5528.438
116	2.024581932	−0.438371147	9.31579965	9.8369	19.4	5587.77
118	2.059488517	−0.469471563	9.41597402	9.8369	19.4	5645.881
120	2.094395102	−0.5	9.51328019	9.8369	19.4	5702.718
122	2.129301687	−0.529919264	9.60768853	9.8369	19.4	5758.23
124	2.164208272	−0.559192903	9.69917027	9.8369	19.4	5812.367
126	2.199114858	−0.587785252	9.78769754	9.8369	19.4	5865.078
128	2.234021443	−0.615661475	9.87324339	9.8369	19.4	5916.317
130	2.268928028	−0.64278761	9.95578176	9.8369	19.4	5966.036
132	2.303834613	−0.669130606	10.0352875	9.8369	19.4	6014.19
134	2.338741198	−0.69465837	10.1117364	9.8369	19.4	6060.735
136	2.373647783	−0.7193398	10.1851051	9.8369	19.4	6105.628
138	2.408554368	−0.743144825	10.2553714	9.8369	19.4	6148.827
140	2.443460953	−0.766044443	10.3225138	9.8369	19.4	6190.293

## Data Availability

The data used to support the findings of this study are included within the article (Table 1).
